# Notes and Comments

**Published:** 1924-03

**Authors:** 


### NOTES AND COMMENTS.

### THE HOSPITALS ASSOCIATION CONFERENCE.

We are able to announce that the annual Conference of the British Hospitals Association will this year be held in London, and that the dates fixed are Thursday and Friday, June 19 and 20. These Conferences have steadily increased in importance until they have become the central event of the year for everyone who is concerned with the management and welfare of hospitals, and in selecting the capital for this year's meeting the Council have made a wise choice. London is the most alluring and, from many points of view, the most convenient place for such a gathering. It is, moreover, the centre of European hospital enterprise, and a Conference within its walls inevitably attracts a degree of public attention far beyond anything that is possible elsewhere. The annual gatherings must necessarily be held, as a rule, in a great provincial town, since it is clearly important to spread first-hand knowledge of the Association's activities over as large a geographical area as possible; yet periodical visits to London are obviously desirable. There could be no more appropriate year than 1924 for such a visit, since the capital will this summer be a place of pilgrimage for myriads of visitors from the Dominions and foreign countries, anxious to see the wonders of that Empire Exhibition which promises to be the biggest thing of its kind that has yet been organised.

### A PARLIAMENT OF HEALTH.

The past month has been notable for the elevation of the Health Committee of the League of Nations from the status of a Convention to that of a constitutional Parliament. Hitherto it has been a merely Provisional body, but it has now become the Executive of the Health Organisation of the League, and enjoys the advantage of entering into the fruits of the six sessions that have already been held. Elsewhere we give some account of the constitution of the Committee and of the work it has in hand. In these February sittings consideration has been given to a number of subjects of high importance to the sanitary welfare of the world. Thus a report has been received dealing with the amounts of opium, cocaine, morphine and such-like dangerous drugs annually needed by medicine in this or that country—information without which it would be impossible to reach an informed conclusion as to the world's legitimate requirements, or to regulate effectively the disastrous traffic in these narcotics. Consideration has been given to a proposed inquiry into the variation in cancer mortality in various countries. Then, for the benefit of the Near East, advice is offered upon the best method of preventing malaria, and there has been discussion upon Holland's excellent suggestion that seaports should be classified in accordance with the sufficiency of their sanitary regulations. This would mean that a ship with a clean bill of health from a "Class A" port could enter another port of the same grade without further examination. This eminently practical suggestion would not only save time and trouble, but, what is much more important, it would put ports with a lower classification upon their mettle and induce them so to improve and modernise their methods that they might fairly aspire themselves to reach the first class. Emulation is a powerful reforming force.

### THE ANCIENT SCOURGE.

The important meeting at the Mansion House in support of the new campaign against leprosy, of which we print a necessarily brief report, has brought prominently before the country the splendid possibility of finally extinguishing this ancient and terrible scourge. In the present state of public finance in England it would be too much to expect that it could raise the £250,000 that is needed to do the work thoroughly, but leprosy is the concern of the whole Empire; in fact, it is much more the concern of India and other distant Imperial lands than of the Mother Country, where the disease is almost unknown. It ought not to be outside the possibilities of the Empire to provide this large sum as it is wanted. But although England knows little of leprosy at first hand, it does, unhappily, know something, and we are sorry to learn that the St. Giles Homes, the only place in the British Isles in which lepers are segregated and treated, is in need of money. A generous donor has promised to give five shillings in the pound of any sum that may be collected for their aid up to £4,000, and we sincerely hope that the Secretary of the Homes, at 10, Old Jewry Chambers, E.C. 2, will speedily be able to report that the money has been subscribed. Let us not forget that cure, and not mere alleviation, appears now to be possible.

### THE INVALID EX-SERVICE MAN.

During the past month two entirely laudable steps have been taken in relation to ex-Service men who are in mental hospitals or are suffering from tuberculosis. Under the provisions of the Royal Warrant men whose mental condition has been decided to be neither attributable to, nor aggravated by war service, ceased to be chargeable to the Pensions Vote one year after the termination of the war—that is to say, September 30, 1922. Put into plain English, this meant that they thereupon became paupers, chargeable to the Union. A state of things was thus created which could not be made comfortably to fit the national conscience, which rebelled against treating as paupers men who did their duty by their country in its hour of trial. These unhappy victims numbered 770, and we rejoice that the Minister of Pensions has seen his way to pay for their maintenance from a special Exchequer grant so that no taint of pauperism can attach to them now or in the event of their recovery. Wisdom and Christian charity have likewise presided over the arrangements that have been made for the active co-operation of the local agencies of the Ministries of Labour and Pensions in the after-care of ex-Service men suffering from tuberculosis on their return home after a course of treatment and training.

These agencies are charged to see that the men's home conditions are satisfactory, and to endeavour to improve them if they are not, and to see that their occupations are suitable. It would be at once cruel and stupid to care for a patient in a sanatorium and then, when he is deemed to be well enough to go away, throw him into the vague, to sink or swim. Care and forethought will save many a life that has been endangered by the hardships of war.

### "RED COTTON NIGHT-CAP COUNTRY."

Sir James Cantlie has been placing a fearful alternative before the average carnal man—whether to risk rheumatism and neuralgia, or to wear a night-cap in a bed through which a warming-pan has made a leisurely and comforting progress. The *homme moyen sensuel* will probably splutter with indignation at being offered so atrocious a choice. He has seen pictures of old ladies wearing frilled night-caps; he may even have seen his own grandmother arrayed in this archaic panoply, and in his wrath he is sadly likely to make scornful remarks about a very distinguished man of science. He is profoundly convinced that, having once " gone out," no earthly power can restore the night-cap to the pillow, although, were he more philosophical and less superior, he might recognise that the deader a fashion is the more certain it is to come back to life. To the weak vessel the warming-pan may offer attractions—he may be incapable of enjoying the glorious masculinity of a deliberate plunge into a cold bed on a chilly night. The warming-pan really does seem to be extinct, save as a wall ornament, killed by the superior attractions and conveniences of the hot-water bottle. In old age there is much to be said for a warm couch, but now every girl goes to bed hugging a hot-water bottle, which is sometimes ungrateful enough to turn traitor. In the end we must all settle for ourselves this solemn question of bed temperature, but the amazing popularity of the hot-bottle is to a great extent a matter of fashion, while the wearing of the cap has been transferred from night to morning, when it is, we understand, highly ornamented and called a boudoir-cap, and is certainly not assumed from fear of rheumatism. Sir James Cantlie dislikes dentists and toothbrushes as much as he admires night-caps and warming-pans. Neither of them, he says, is mentioned in the Scriptures. For the matter of that, there is not much about doctors either in those writings.

### THE EXCHANGE OF MEDICAL OFFICERS.

Eight-and-twenty foreign medical officers from nineteen different countries are at present in England studying our public health administration. They have been enabled to come to us by that Health Section of the League of Nations which has already accomplished such admirable work in the internationalising of health, and before long representative Medical Officers of Health will go from us to other countries to study their methods in turn. Those who have already taken part in these valuable interchanges have found that England by no means possesses a monopoly of what may be called health enterprise. We may have shown the way, but very often our instruction has been bettered, and if the foreign medical officer may still learn from us, we can also learn from him. Our visitors—they are not the first company to reach these shores—will be here until April, and their itinerary is so large and so leisurely that they will enjoy opportunities of learning in considerable detail the nature of the work we are doing. Our problems are not always theirs, but the dealing with one problem often throws light upon others, and, in any case, both we and they can realise that in a very wide sense in seeking and ensuing the means to health we are cultivating that truest internationalism which is founded upon an enlightened nationalism.

### A LITTLE QUESTION OF HERALDRY.

ST. THOMAS'S HOSPITAL has among its students a heraldic enthusiast who has had the temerity to declare that the arms used by the hospital are " bogus," or " spurious "—there seems to be some doubt about the precise phrase, but, whatever it may be, it is highly derisory. He collected enough money to pay the Herald's College fee for a new grant of arms—the cost is, we believe, about £76—and then asked the permission of the Governors to apply for it. They failed to see the necessity for anything of the kind; they have used the existing arms since time of Edward VI., which is quite a good period of prescription. The heraldic cognisance of St. Thomas's—a mélange of the arms of the City of London, the lilies of France, and the Tudor rose—is the same as that used by the other Royal Hospitals—Christ's Hospital and Bridewell. When Edward VI. granted the Charter to the Royal Hospitals in 1552 they were all placed under the management of the Aldermen of the City of London, and they obviously took the City's heraldry as the basis of the arms they adopted. It may well be that they are not registered at Herald's College. The purists declare that everything not so registered is " bogus," but since there are many old families with unimpeachable arms of which the College has no record, St. Thomas's is in good company. We confess to some curiosity about the ultimate destination of the £76. The Emperor Pedro of Brazil built a hospital with the proceeds of sales of titles of nobility, and placed upon its front the inscription: " Human vanity to human suffering." No doubt the Governors of St. Thomas's would be able to think out an equally appropriate inscription for some useful hospital appliance bought with the money originally destined for a bit of illuminated parchment from Queen Victoria Street.

### SUGGESTION AND THE TRACTOR.

DR. JAMES J. WALSH, Professor of Physiological Psychology in New York, has lately reviewed the history of the relief of pain from the time of I-em Hetep, of one of the earliest dynasties of Egypt, to that of Coué. Apart from medicinal means for the relief of pain, we do not seem to have advanced greatly, and the well-known aphorism of Hippocrates that one pain drives out another as well as one nail may drive out another, is as true today as it was 2,500 years ago. Many years ago Dr. Elijah Perkins, of Connecticut, made a pair of metallic wands, or "tractors," the ends of which were placed in contact, the patient being stroked with the other ends. If there was a pain in the back, the stroking was done from the spine outwards, and gradually the pain was induced to shift, and then to leave entirely. He was evidently thoroughly convinced that he had made a great discovery, and he undoubtedly cured large numbers of people. Nearly all who went to him were relieved. He proclaimed that his tractors could not only cure, but also prevent, disease. While an epidemic was in progress in Philadelphia, he went there to arrest it by "tractoration," but he caught the disease and died of it. Dr. Perkins's son carried the treatment to England where hundreds of people pronounced themselves cured in the very first year. This number mounted to thousands in the following year, and after three years the younger Perkins was quite sure that there were nearly a million people who had benefited by the tractors. A "Perkinian" institute was established for the benefit of the poor under the patronage of Lord Rivers, "just as we are to have a Coué clinic here in America." Professor Walsh concludes that the history of medicine is more largely composed of the suggestive cures than of any others, and that suggestion has probably relieved more pain and cured more disability, consciously or unconsciously, than any other remedy.

### BLUNDERBUSS PHARMACY.

We may laugh at the twelve-ingredient prescriptions of our fathers and grandfathers, but their polypharmacy was as nothing to that of the physicians of the seventeenth century, as we may see from a glance at a Swedish pharmacopœia published in 1686 by the "Collegium Medicum" of Sweden. This pharmacopœia contained a full eight hundred different prescriptions, and some of the mixtures were mixtures indeed! "Tinctura bezoartica ex herbis," for example, rejoiced in seventy-nine different ingredients, among which was the teriak of ancient history. In the time of Mithridates teriak was a comparatively modest compound of only some fifty odd substances, but by the seventeenth century the prescription had grown to sixty-six ingredients, which included the flesh of snakes (preferably from Italy), balsams, spices, such as pepper and ginger, squills, myrrh, opium, rhubarb, and much else. This valuable concoction took considerable time to mature; it was allowed to ferment for two years, and it was not till two to six years later that its highest potency was reached. Though it is tempting to poke fun at the contents of a seventeenth century pharmacopœia, it is safe to prophesy that a century or two hence the present edition of the "British Pharmacopœia" will be regarded as a quaint collection of drugs, the efficacy of many of which has solely depended on suggestion.

### THE DOCTOR'S BILL.

One of the hardships which the National Health Insurance Act was designed to meet was that of paying the family doctor's bill after the illness of the breadwinner when, generally, he was least able to meet it. It will be remembered that provision was made in the scheme of the Act for voluntary insurance and that, after the experience of a year or two, this was dropped as it was not a success. The Essex general practitioners have now started a scheme of their own, under which those who are eligible can pay weekly subscriptions and can call in the services of the doctor of their choice when they fall ill without having to worry about the bill. The service is limited in scope as in the case of the National Health scheme; it does not include confinements or specialist services. But it is more expensive, as is inevitable in a scheme in which participants can exercise the option of coming in and, in general, may be expected to exercise that option against the doctors—that is, by coming in when they think that they are likely to get some benefit out of it. There is a provision in the scheme to meet this difficulty to some extent. Those who are admitted after the scheme has been in operation six months will have to pay 2s. 6d. for each attendance up to four if they require treatment within six weeks of joining the scheme. This is in addition to the weekly subscriptions, which are 4d. for an individual over 16, or 6d. for husband and wife jointly, and 2d. for each child up to a maximum of 6d. for all the children of one family. The persons who are eligible are those who "belong to the wage-earning classes or who are unable to pay the ordinary medical charges." The promoters are wise in not defining eligibility by reference to an income limit; this in practice would prove to be inquisitorial and unworkable. At the same time the Committee have an entire discretion whether to admit a member, and will exercise this discretion, it is understood, so as to exclude those persons who may reasonably be supposed to have an income of more than £250 a year. The working of the scheme will be followed with a good deal of interest.

### PROTECTIVE EYE-GLASSES FOR MANUAL WORKERS.

In a paper published by Dr. Schou in the official Annual Report on the health of Copenhagen in 1922, he urges the necessity for manual workers in certain occupations to wear protective glasses far more frequently and systematically than is the case at present. Among the 13,000 patients attending the Eye Polyclinic in Copenhagen in the period 1910-1919 there were about 1,300 suffering from foreign bodies in the eyes. Dr. Schou points out that, according to the statistics of other oculists, about 40 per cent. of the eye casualties among manual labourers are due to foreign bodies, whereas this is the case with only about 1 per cent. of intellectual workers. It would be well if in certain industries entailing special risks of injuries to the eyes, the wearing of protective eye-glasses were to be enforced. Unfortunately, they are unpopular with both employers and employees, as the wearing of glasses is supposed to reduce the output of work on the one hand, and to have a depressing effect on the other. The same objections might, of course, be made to the wearing of gas masks in warfare, and if we go further afield, objections can always be raised to insurance against any kind of loss; we do not pay burglary, fire and other premiums merely for the pleasure of enriching insurance companies. It is probably a safe prophecy that, in the near future, the wearing of protective eye-glasses in certain industries will not only be compulsory, but will be taken as a matter of course.

### INNOCENT MOUTHPIECES.

The telephone mouthpiece leaves the Court without a stain upon its character. Once more aspersions have been cast upon it, and once more the Postmaster-General assures those who are afraid of being infected with tubercle bacilli by using public telephones that bacteriological tests have shown that there is no danger whatever. It is easily possible to be much too nervous about matters of this kind, and if nothing dreadful happens from handling coin and Treasury notes—a much more frequent and general operation than telephoning—there is little need to be scared about telephone mouthpieces. Neither Exchange operators nor bank clerks die in their thousands from the risks of their occupations, and the nervous people who put gauze or paper protectors upon their telephone mouthpieces may save themselves the trouble. In America, where the telephone is in much more common use than in England, careful experiment has also had satisfactory results. To many people a little scare is so comforting that it seems unkind, and almost brutal, to rob them of what they easily come to regard as part of their birthright. Yet the harder an error is knocked on the head the harder it flaps its wings and the louder it cackles. We expect, therefore, yet to meet the tuberculous telephone mouthpiece in many a call-office and " on " many a railway station—to use the inelegant and ungrammatical language of the Postmaster-General.

### CHEAP RATTLESNAKES.

A recent number of the *Pharmaceutical Journal* contains an interesting note on the active Brazilian trade carried on in live snakes for the direct purpose of preparing serum to be injected as an antidote for snake-bite. In earlier days it appears that dwellers up-country used to send to the institute at Butantan, São Paulo, as many snakes as they could catch and receive, in return, supplies of anti-serum. But in spite of the prevalence of snakes in the country, sufficient was not obtained in this way, and now regular prices are paid and snake-hunting on a large scale encouraged. Nevertheless, a reward of about eighteenpence for catching and sending in alive a full-grown rattlesnake hardly suggests that the profession will be overcrowded.

### THE WIDOW'S CLAIM.

One of the chief causes of undeserved poverty, Miss Eleanor Rathbone has been pointing out in the *Times*, is the premature death of the wage-earner, and for this reason she has been urging the claim of the widow with young children to a moderate pension. Poor Relief, rightly or wrongly, carries with it a stigma, and, roughly speaking, only 28 per cent. of such widows apply for it. Then the scale of relief varies extraordinarily. In 1919 a Yorkshire Board gave 63s. per week to a widow with six children ; a Midland Board gave 10s. a week to a widow with seven. It is obviously impossible for a poor widow to bring up a family without some outside assistance, and the task of somehow scraping together the wherewithal for existence is too heavy for any woman's strength. The effect upon her health is bound to be disastrous ; in fact, vital statistics show that the widow's chance of life is worse than that of the wife or spinster. And if common humanity did not demand that something should be done to alleviate her lot, the national welfare demands that her children should have an opportunity of growing up healthy and happy. The lack of a father is a severe handicap to a boy or girl, the former particularly, and the mother needs all the help the State can give her if her children are to become useful citizens and not inhabitants of our prisons or infirmaries. The country's burden is just now particularly heavy, but where health and efficiency are concerned we can ill afford to economise, and the recent debate on this subject in the House of Commons elicited the announcemeut that the Government are considering the subject in all its details.

### TAX-FREE BEQUESTS: A SOLUTION?

To meet the objections officially made that bequests to hospitals should be exempt from Legacy Duty, the Lancashire Voluntary Hospitals Committee propose that the duty should be collected by the Treasury, as hitherto, and then transferred to the Voluntary Hospitals Commission for distribution to necessitous hospitals on a plan approved by the Treasury. This would meet the difficulty that, as many charitable bequests are made free of duty, the proposed exemption would benefit not the charities but the residuary legatees. Mr. J. J. Courshott the Chairman of the Lancashire Local Committee, who signs its report, points out that the proposal to ear-mark a particular source of revenue for a particular purpose is already adopted in regard, for instance, to the appropriation of taxes on motor vehicles for the maintenance of roads. Moreover, Parliamentary action has already placed hospitals in a privileged position through the Commission that it provided to distribute the Government grant, and therefore it could resist consistently similar applications from other charities.

### SET A THIEF ——.

It is well known that depredations by insects are kept within bounds by the activities of animals and birds. But other insects are frequently of great use. In this country a condition of relative stability has been reached in the average prevalence of the various insect types, and to disturb this balance it is necessary to introduce new varieties from outside. An instance of such controlled disturbance is, we learn, about to be attempted through the Ministry of Agriculture's Laboratories. A fly obtainable from France is found to be specially antagonistic to the woolly aphis, or American blight, and to other kinds of " grain fly." Thousands of this fly's cocoons have been artificially reared and are to be distributed throughout the country in the hope of permanently establishing the insect, and so bringing about a wholesale reduction in the prevalence of those aphides which cause such heavy horticultural and agricultural loss.

